# Inter-community behavioural variation confirmed through indirect methods in four neighbouring chimpanzee communities in Cantanhez NP, Guinea-Bissau

**DOI:** 10.1098/rsos.211518

**Published:** 2022-02-23

**Authors:** Joana Bessa, Dora Biro, Kimberley Hockings

**Affiliations:** ^1^ Department of Zoology, University of Oxford, Oxford, UK; ^2^ Centre for Research in Anthropology (CRIA NOVA FCSH), Lisbon, Portugal; ^3^ Department of Brain and Cognitive Sciences, University of Rochester, Rochester, NY, USA; ^4^ Centre for Ecology and Conservation, College of Life and Environmental Sciences, University of Exeter, Penryn, UK

**Keywords:** animal culture, behavioural variation, *Pan troglodytes verus*, anthropogenic habitat

## Abstract

Culture, while long viewed as exclusively human, has now been demonstrated across diverse taxa and contexts. However, most animal culture data are constrained to well-studied, habituated groups. This is the case for chimpanzees, arguably the most ‘cultural’ non-human species. While much progress has been made charting wild chimpanzees' cultural repertoire, large gaps remain in our knowledge of the majority of the continent's chimpanzees. Furthermore, few studies have compared neighbouring communities, despite such comparisons providing the strongest evidence for culture, and few have studied communities living in anthropogenic habitats although their culture is in imminent danger of disappearing. Here we combine direct, indirect and remote methods, including camera traps, to study, over 2 years, four unhabituated neighbouring chimpanzee communities inhabiting human-impacted habitats in Cantanhez NP, Guinea-Bissau. From traces collected during 1089 km of reconnaissance walks and 4197 videos from 56 camera trap locations, we identified 18 putative cultural traits. These included some noteworthy novel behaviours for these communities, and behaviours possibly new to the species. We created preliminary behavioural profiles for each community, and found inter-community differences spanning tool use, communication, and social behaviour, demonstrating the importance of comparing neighbouring communities and of studying previously neglected communities including those inhabiting anthropogenic landscapes.

## 1. Introduction

Animal culture, defined as patterns of behaviour that are group-typical and transmitted, at least in part, through social learning [1], is argued to be a source of adaptive behaviour: individuals can more readily discover the solution to a problem if they attend to or copy the behaviour of other individuals facing the same problem, allowing them to better exploit their natural and social environment. Furthermore, cultural traditions can influence evolutionary rates and trajectories by homogenizing behaviours within a population and allowing rapid changes in a population's behavioural repertoire [3]. Overall, the study of animal culture can inform researchers of different ways animals adapt to their environment (e.g. foraging techniques, migratory patterns, communication of information), and might be a valuable tool when planning conservation strategies [2,4–6]. The first evidence for animal culture was described over 70 years ago where the transmission of new behaviour (milk-bottle opening) by titmice (*Parus* sp.) was tracked across the United Kingdom [7]. Soon after, novel foraging behaviours that spread through kin networks were found in Japanese macaques (*Macaca fuscata*) (e.g. sweet potato washing) [8], and regional birdsong dialects were discovered in white-crowned sparrows (*Zonotrichia leucophrys)* [9]*.* Since then, evidence of animal culture has been identified in numerous taxa, from fish to meerkats, cetaceans and apes (e.g. [10–14]), both in the wild and in captivity.

Multiple approaches to studying animal culture have been proposed. Among these, the one most widely used in the wild to date is the ethnographic method [15] or ‘method of exclusion’ [16] that identifies culture by ruling out possible ecological or genetic explanations for inter-group behavioural variation documented. A paradigmatic example of this method was the first large-scale chimpanzee cross-population study that extracted 39 candidate behavioural traits from six habituated chimpanzee communities in West and East Africa [17]. These included habitual or customary behaviours that could not be explained by ecological or genetic differences between populations, hence by exclusion were considered cultural variants. Some experimental methods rely on a similar rationale: for example, translocation experiments, where individuals are moved from one population to another or whole populations are exchanged between sites [10,18], seek to establish whether existing behavioural variation is more likely to be traditional than due to genetic or environmental influences. Nonetheless, many argue that methods of exclusion, if rigorously applied, might erroneously reject cases of animal culture (e.g. [2,15,19]), and in fact, ecology, genetics and social learning are inexorably interlinked and can all influence, to some degree, behavioural variation [19,20]. For more direct demonstrations of cultural processes, some researchers have employed elegant field experiments where new resources or information are artificially introduced into a wild community to observe, in real time, their diffusion through social learning (e.g. [21,22]). Nonetheless, although these studies allow us to confirm that new behaviours can spread through groups through social learning, they do not inform us about the nature and spread of naturally occurring behavioural variation [11]. Statistical approaches try to tackle this problem: for example, network-based diffusion analysis (NBDA) has been used to study the transmission of newly invented behaviours in chimpanzees [23], humpback whales [14] and bottlenose dolphins [24]. While powerful when the right data are available, one limitation of the NBDA approach is that it requires long-term data collection on the same population, as well as some degree of luck in witnessing the natural emergence and transmission of a novel behaviour.

While large-scale ethnographic studies have given us valuable insights into species-level variation in putatively cultural behaviours [17,25], it has been suggested that more compelling evidence for culture might come from studying the same subspecies (e.g. [12,19]). For example, while all four subspecies of *Pan troglodytes,* but not all known communities, engage in army ant (*Dorylus* spp.) dipping, there is variation in the technology employed even among different communities of the same subspecies [26]. Some of this variation can be linked to the aggressiveness of the ant species exploited, but in some cases, such an ecological account does not fully explain the variation found [5,27]. Therefore, many have recently suggested that the study of neighbouring communities might be the most informative approach to studying animal culture in the wild (e.g. [11,12,28,29]): comparing communities where groups broadly face the same ecological constraints and individuals migrate between communities, make ecological or genetic explanations for behavioural variation less likely or important compared to a cultural explanation. Illustrating this approach, Luncz *et al.* [12] compared the selection of wooden and stone hammers for coula (*Coula edulis*) nut-cracking in three habituated neighbouring chimpanzee communities in Tai National Park, Ivory Coast. Even though these neighbouring communities inhabit the same forest habitat with minimal ecological variation between their home ranges, the study showed that there was marked inter-community variation in hammer size and raw material preferences [12]. Similarly, Thornton *et al.* [11] have shown that neighbouring groups of meerkats show consistent differences in their time of emergence from their sleeping burrows, despite overlaps in burrow use and extensive gene flow between groups. In sum, it is clear that different methods have their pros and cons and their feasibility is dependent on species, environment, financial and ethical constraints, among others [6], but many of the examples here described emphasize the need for more comparative studies of behavioural variation at a local scale, both in terms of the presence/absence of specific behaviours across communities and their detailed descriptions that may reveal subtle variation in form.

In chimpanzees, arguably the most ‘cultural’ among non-human species, much effort has focused on building a comprehensive catalogue of the species' cultural repertoire [17,25]. Yet, while much of the species’ range has been studied [5,30–35], new behaviours and behavioural variants keep being identified, suggesting that gaps still remain to be filled. At the same time, increasing human disturbance, and consequent fragmentation, degradation and change of habitats and available resources, are suggested to have the potential to bring about both the loss of existing behavioural variants [5] and their modification as an adaptive response to environmental changes [4,32,36]. This highlights the importance of surveying communities inhabiting anthropogenically impacted areas. Specifically, little is known about the westernmost populations of the species' distribution. This means that not only are we lacking detailed information about potential regional cultural variation but we may have a limited time in which to chart it: behaviours may go extinct, and we may lose our ability to document how chimpanzees respond to life in the Anthropocene [37].

The present study aims to fill these gaps by being the first to explore cultural variation between neighbouring chimpanzee communities inhabiting Cantanhez National Park (CNP), Guinea-Bissau. CNP is thought to be home to approximately 10–12 chimpanzee communities [38] inhabiting a mosaic of habitats. Bessa *et al.* [39] studied the feeding ecology of the chimpanzee community of Caiquene-Cadique in CNP for nine consecutive months, where apart from the use of leaf sponges, a universal chimpanzee behavioural trait, no other type of tool use was confirmed (unpublished data). A recent study, however, has found evidence of honey-dipping tools in other CNP communities [40]; Bessa *et al.* [39] also suggest, based on indirect data (i.e. traces such as accumulation of snail shells, use-wear marks in wooden anvils and bite marks in soft tissue of discarded snails), the possibility of the Caiquene-Cadique chimpanzees cracking giant African snails (*Achatina achatina)* against wooden anvils and eating them (a behaviour first described in a recent study of Bili-Uéré's (DRC) chimpanzees, though direct evidence is yet to be found [41]). Additionally, a recently discovered behaviour—accumulative stone throwing [34,42]—has also been confirmed in Boé National Park, Guinea-Bissau. Overall, preliminary work indicates the presence of a potentially unique cultural profile for the poorly studied Guinea-Bissau chimpanzees.

We collected behavioural data on four unhabituated neighbouring chimpanzee communities in central CNP. Due to the communities' close proximity to local people, we chose not to habituate chimpanzees, therefore any direct observation was merely opportunistic, and the majority of data collection relied on a combination of indirect methods and camera traps. As such, our aim was also to demonstrate the feasibility of using this combination of methods to achieve our three primary goals: (1) to document new behaviours for the studied communities, expanding our to-date limited knowledge of their behavioural repertoire; (2) to identify behavioural variation among communities; and (3) to contribute to continental-level comparisons in the ever-growing chimpanzee behavioural repertoire. Our indirect data collection methods (see below) were particularly suitable for behaviours that leave trace evidence (extractive tool use, resource consumption), while the camera trap data were our principal source of information for behaviours that are more ephemeral/do not leave traces (communication and display, social behaviour). We structure our results according to these categories.

## 2. Methods

### 2.1. Study site

**Figure 1.**
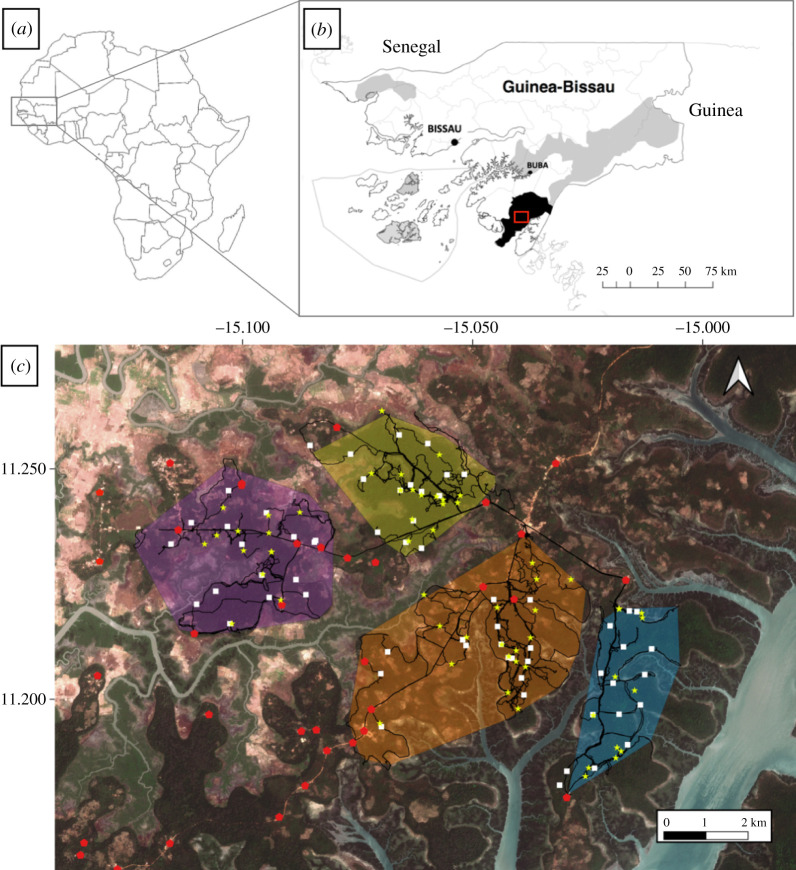
Locations of research sites and study communities' core home ranges. (*a*) Map of Africa showing the location of Guinea-Bissau. (*b*) Map of Guinea-Bissau showing the locations of CNP (black) and other protected areas (light grey). (*c*) Aerial image showing the locations of the four study sites in CNP. The core home ranges of the four chimpanzee communities, minimum bounding polygons estimated from direct and indirect chimpanzee data points, are illustrated in different colours: [CC] Caiquene-Cadique (purple), [LA] Lautchandé (yellow), [MA] Madina (orange) and [CB] Cambeque (blue); the minimum bounding polygon for the core home range of this chimpanzee community provides only a rough estimate given the paucity of direct and indirect data points available. Black lines indicate recces walked, red pentagons correspond to locations of villages and other human settlements, white squares correspond to 50 × 50 plots, and yellow stars correspond to camera trap locations. Sentinel-2 imagery was downloaded from the Sentinel Hub, Sinergise Ltd (https://www.sentinel-hub.com/). All maps were created using QGIS version 3.12 (https://www.qgis.org).

CNP is located in the Tombali region of south-west Guinea-Bissau. CNP has an area of 1057 km^2^ and is a mosaic of settlements, agricultural fields, sub-humid forest, secondary forest, palm groves, mangrove and savannah. Recent work has revealed that a large percentage of forest cover remains across the habitat matrix, but these forest areas are highly fragmented and interspersed with cultivated land. Areas cleared for agriculture often have remnant forest edges that together with fallow land and orchards can work as corridors for some wildlife [38] (figure 1). Data collection took place in four neighbouring unhabituated chimpanzee communities at CNP: Caiquene-Cadique, Lautchandé, Madina and Cambeque, over the course of 23 consecutive months (February 2017–December 2018). We identified different communities based on previous research, including genetic studies, local knowledge and the presence of natural and anthropogenic barriers (e.g. villages, roads, rivers and estuary branches that form small peninsulas) [38,39,43–46]. The size of each community's ranging area was estimated using minimum convex polygons: Caiquene-Cadique 14.8 km^2^, Madina 19.0 km^2^, Cambeque 7.1 km^2^ and Lautchandé 8.4 km^2^. Additionally, previous work had estimated the minimum community size in Caiquene-Cadique as 49 individuals (Ramon, personal communication) and Madina as 48 individuals [44]; for Lautchandé or Cambeque we did not possess sufficient information to estimate community sizes.

### 2.2. Data collection

As the chimpanzees of CNP are unhabituated to researchers, a combination of direct, indirect and remote data collection methods was employed. To ensure that data collection was systematic and comparable to that of other study sites across Africa, methods were adapted from the Pan African Programme guidelines [5,47] and are explained in detail below. Study efforts across the four sites are summarized in table 1.

**Table 1.** Summary of study effort at each of the study sites, in Cantanhez National Park in Guinea-Bissau. All relevant resources were confirmed in all studied communities apart from *Melipona* sp., only confirmed in Cambeque and Madina. Information is provided on number of recces and kilometres (km) walked; camera trap (CT) number, operational period (first day and last day active) and total number of days active and functional; total number of videos recorded that contained chimpanzees; estimated ranging area; indirect data points collected; number of confirmed behaviours; behaviours and their categorization (C, presence confirmed through indirect data; C*, presence confirmed through direct evidence (video or observation); U, unconfirmed) at the four study sites in CNP. Additionally, the total number of indirect data (I) and camera trap videos (V) of each behaviour is given for each community after the behaviour categorization (I,V).

		chimpanzee community			
		Caiquene-Cadique	Cambeque	Lautchandé	Madina
recces	no. of recces	48	49	48	50
	distance walked (km)	230.8	260.4	236.6	361.5
camera trap deployments	no. of CTs	12	11	15	18
	first CT day	05/04/2017	07/03/2017	06/05/2017	20/02/2017
	last CT day	08/12/2018	22/11/2018	10/12/2018	04/12/2018
	total CT days	2367	2391	2109	2828
	total no. chimpanzee videos	2254	828	468	647
data	estimated ranging areas (km^2^)	14.8	7.1	8.4	19.0
	no. of indirect traces	538	230	230	749
	no. of confirmed behaviours	17	14	9	18
behaviour	fluid-dip	U (0,0)	U (0,0)	C (1,0)	U (0,0)
	honey-dip	U (0,0)	U (0,0)	U (0,0)	C (2,0)
	honey-dip large stingless bees	U (0,0)	C* (63,2)	U (0,0)	C (1,0)
	honey-dip small stingless bees	U (0,0)	C (38,0)	U (0,0)	C* (100,8)
	leaf-sponge	C (6,0)	C (6,0)	C (5,0)	C* (4,3)
	honey-feed, no tools	C* (10,4)	C (3,0)	C (5,0)	C (2,0)
	mangrove-eat	C* (6,7)	C (2,0)	U (0,0)	C (7,0)
	saltwater-drink	C* (0,4)	U (0,0)	U (0,0)	C* (0,4)
	aimed-throw	C* (0,4)	U (0,0)	U (0,0)	U (0,0)
	branch-drag	U (0,0)	U (0,0)	U (0,0)	C* (0,1)
	branch-shake	C* (0,10)	C* (0,2)	C* (0,2)	C* (0,8)
	buttress-drum	C* (15,624)	C*(8,110)	C* (11,141)	C* (32,171)
	ground-slap	C* (0,3)	C* (0,2)	U (0,0)	C* (0,1)
	ground-slap knuckles	C* (0,1)	C* (0,1)	U (0,0)	C* (0,1)
	leaf-clip, fingers	C* (0,33)	C* (0,1)	C* (0,3)	C* (0,4)
	leaf-clip, mouth	C* (0,34)	C* (0,4)	C* (0,7)	C* (0,19)
	leaf-drag	C* (0,11)	U (0,0)	C* (0,2)	U (0,0)
	leaf-pull, finger	C* (0,10)	C* (0,1)	U (0,0)	C* (0,5)
	leaf-pull, mouth	C* (0,2)	C* (0,1)	C* (0,1)	C* (0,3)
	rain-dance	C* (0,13)	U (0,0)	U (0,0)	U (0,0)
	raspberry	C* (0,3)	C* (0,1)	U (0,0)	C* (0,14)
	food-share	C* (0,7)	U (0,0)	U (0,0)	C* (0,13)

#### 2.2.1. Indirect data

Reconnaissance walks (‘recces’, which followed given compass bearings while walking the path of least resistance [47]) were conducted at each study site by following chimpanzee paths and forest trails that covered as many different habitat types as possible. This method was chosen over systematic transects in order to minimize disturbance to an already highly fragmented habitat and to avoid opening up new trails for hunters. Recces were walked in rotation between communities, from February to July 2017 and from February to July 2018 spanning across the second half of the dry season and the first half of the wet season (see table 1 for number of recces and total distances covered per community). During recces, data on direct encounters (i.e. group size, composition, location and behaviours) and indirect signs of chimpanzee presence and behaviour, including faeces, feeding traces, nests, tool-use sites and foot and knuckle prints, were collected. These data were used to estimate chimpanzee core ranging areas and to find the best locations to set up camera traps. Any plant and animal species associated with chimpanzee behaviour (e.g. feeding traces; tools used in extractive foraging) that were not identified *in situ* were collected for later identification by local people or, when necessary, by consulting the relevant literature (see [48,49]). Every behaviour that was identified in one community was added to a list of candidate behaviours for inter-community variation, and efforts were made to confirm the presence of each in all of the other communities.

#### 2.2.2. Remote data

Camera traps (Bushnell Trophy Cam HD Aggressor No-glow) were set up at 56 different locations in CNP (see table 1 for a more detailed breakdown). Camera traps were motion triggered and recorded 1-minute videos, and were set up in places habitually used by chimpanzees and where (1) tools had been found and/or tool use behaviour was expected to take place (e.g. stingless bee hives; natural water sources) and (2) where there was evidence of habitual presence of chimpanzees and where other behaviours of interest could occur (e.g. trees with large buttress roots with clear signs of wear). To maximize the chances of capturing behaviours of interest, some cameras were moved during the study to account for seasonal changes in chimpanzee ranging patterns, and to capture previously undocumented behaviours.

#### 2.2.3. Resource availability data

During recces, data on the presence of specific resources were recorded *ad libitum*: nut-bearing trees, movable stones, beehives, termite and army ant nests and trails and giant African snails. These resources were selected since they are associated with specific putative cultural behaviours, mostly tool use, in other studied communities. Additionally, fifteen 50 × 50 m plots were established at random locations in each of the four communities' ranging areas to assess the presence/absence of the same resources. When movable stones were found they were tested for hardness and sturdiness by hitting them twice with significant force against a wooden or stone substrate; if the stone did not fragment it was considered potential tool raw material [47].

### 2.3. Data analysis

We compiled a list of behaviours present based on the data collected (including camera trap footage) and categorized observations as either species-typical behaviours (i.e. present universally across all habituated chimpanzee populations), or putatively cultural (i.e. present at some or all of our study sites but not across the whole species range, or present at at least one, but not all of our study sites). The categorization was done based on previously published data on chimpanzee culture and behavioural variation (following [5,17]). Definitions for each behaviour are given in table 2 (adapted from [5,17,50]). Given that these are previously unstudied and unhabituated communities, data were insufficient to categorize behaviours as customary (occurs in all or most members of at least one age–sex class) or habitual (not customary but seen repeatedly in several individuals), as defined in [25]. Therefore, the following categories were used to describe our knowledge of the prevalence of each behaviour: Confirmed (C)—the behaviour was clearly identified in the community; Unconfirmed (U)—the behaviour was not yet recorded in the community, but this may be explained by insufficient observation opportunities.

**Table 2.** Definition of behaviours recorded in the present study that show potential variation among study sites (adapted from Whiten *et al*. [17] and Nishida *et al*. [50]).

behaviour	definition
extractive tool use	
fluid-dip	manufacturing a probe from a twig to extract fluid.
honey-dip	manufacturing a probe from a twig, to extract honeybee (*Apis mellifera*) honey from nest.
honey-dip large stingless bee forest	manufacturing a probe from a twig, to extract large stingless bee honey (*Melipona* sp.) from nest, generally found in open secondary forest.
honey-dip small stingless bee mangrove	manufacturing a probe from a twig, to extract small stingless bee honey (*Meliplebeia* sp.) from nest, generally found in mangroves.
leaf-sponge	bundling leaves/vegetation, chewing or folding, to collect water and squeeze into the mouth.
resource consumption, feeding and habitat use	
Honey-feed, no tools	feeding on bee honey without a tool, employing snatch and run approach.
mangrove-eat	collecting salty leaves of *Avicennia germinans* (found exclusively in mangrove areas) from tree, either ingesting or chewing and spitting out (wadge).
saltwater-drink	drinking mangrove salt water that collects in puddles.
communication and display	
aimed-throw	aiming and throwing of object.
branch-drag	dragging a large branch as part of a display.
branch-shake	shaking of branch, producing a conspicuous sound, prior to a buttress-drumming display.
buttress-drum	beating/drumming with hands or feet on buttress or trunk of a tree, normally preceded by pant-hoot vocalization.
ground-slap	striking substrate with open hands/feet or alternate hands/feet during display, sometimes followed by pant hoot vocalization.
ground-slap, knuckles	as above, but substrate is struck with the knuckles instead of open hands.
leaf-clip, fingers	ripping apart of one or more, normally dried, leaves from the ground using the thumb and index fingers, one by one, producing a conspicuous and distinctive ripping sound. Typically precedes buttress-drumming display.
leaf-clip, mouth	as above, but clipping is performed with the mouth. Typically precedes buttress-drumming display.
leaf-drag	walking forward fast quadrupedally with head down and shoulders hunched, while pushing dry leaves with hands and feet, producing distinctive sound. Sometimes performed before and/or after buttress drumming.
leaf-pull, fingers	pulling of leaves, one by one, from a shrub or a twig, with index finger and thumb. Typically precedes buttress-drumming display.
leaf-pull, mouth	as above, but pulling is performed with the mouth. Typically precedes buttress-drumming display.
rain-dance	performing vigorous charging displays at the start of heavy rain. May include slow as well as rapid charges, and may involve a variety of display patterns (e.g. ground slap, branch drag, branch shake, throw).
raspberry	producing a spluttering sound by pressing air and saliva through lips.
social behaviours	
food-share	feeding by two or more individuals simultaneously on an item of food obtained by one of the individuals. Theft is excluded.

## 3. Results

During the study period a total of 1089 km were walked over 195 recces, and camera traps were set up in 56 locations, for a combined total of 9695 days, yielding a total of 4197 videos of chimpanzees (see electronic supplementary material, table S1). During recces, 1747 indirect chimpanzee traces were recorded, including 204 extractive stick tools. For detailed information on study effort and data collected in each community, see table 1.

Our surveys of resource availability revealed the following results. At all sites the availability of three species of nut-producing trees (*Elaeis guineensis, Detarium senegalense* and *Parinari excelsa*) was confirmed. Termites (*Macrotermes* sp. and *Cubitermes* sp.) were present at all study sites, as were army ants (*Dorylus* sp.). At other chimpanzee research sites, these resources are exploited with the use of tools (i.e. nut-cracking and termite and ant fishing/dipping) (see [25]). Nonetheless, no evidence of tool-assisted nut-cracking or insectivory was confirmed for CNP; additionally, a previous study that analysed hundreds of faecal samples in Caiquene-Cadique found no macroscopic evidence of insectivory [39]. Movable stones were present in all CNP communities but infrequently encountered. The stones found were fragile and broke easily, and no evidence of their use was found. Giant African snails (*Achatina achatina*) were present in all CNP communities’ home range, nonetheless no conclusive evidence (e.g. direct observation) of the exploitation of snails was found. Honeybees (*Apis mellifera*) were present at all five study sites and a species of stingless bee (*Meliplebeia* sp.) was confirmed for all four CNP sites. A second species of stingless bee (*Meliponula* sp.) was only confirmed at Cambeque and Madina. Tool-use evidence as well as trace evidence of discarded honeycombs with teeth marks confirmed that each CNP community fed on at least one of the different types of honey (see [40]).

**Figure 2.**
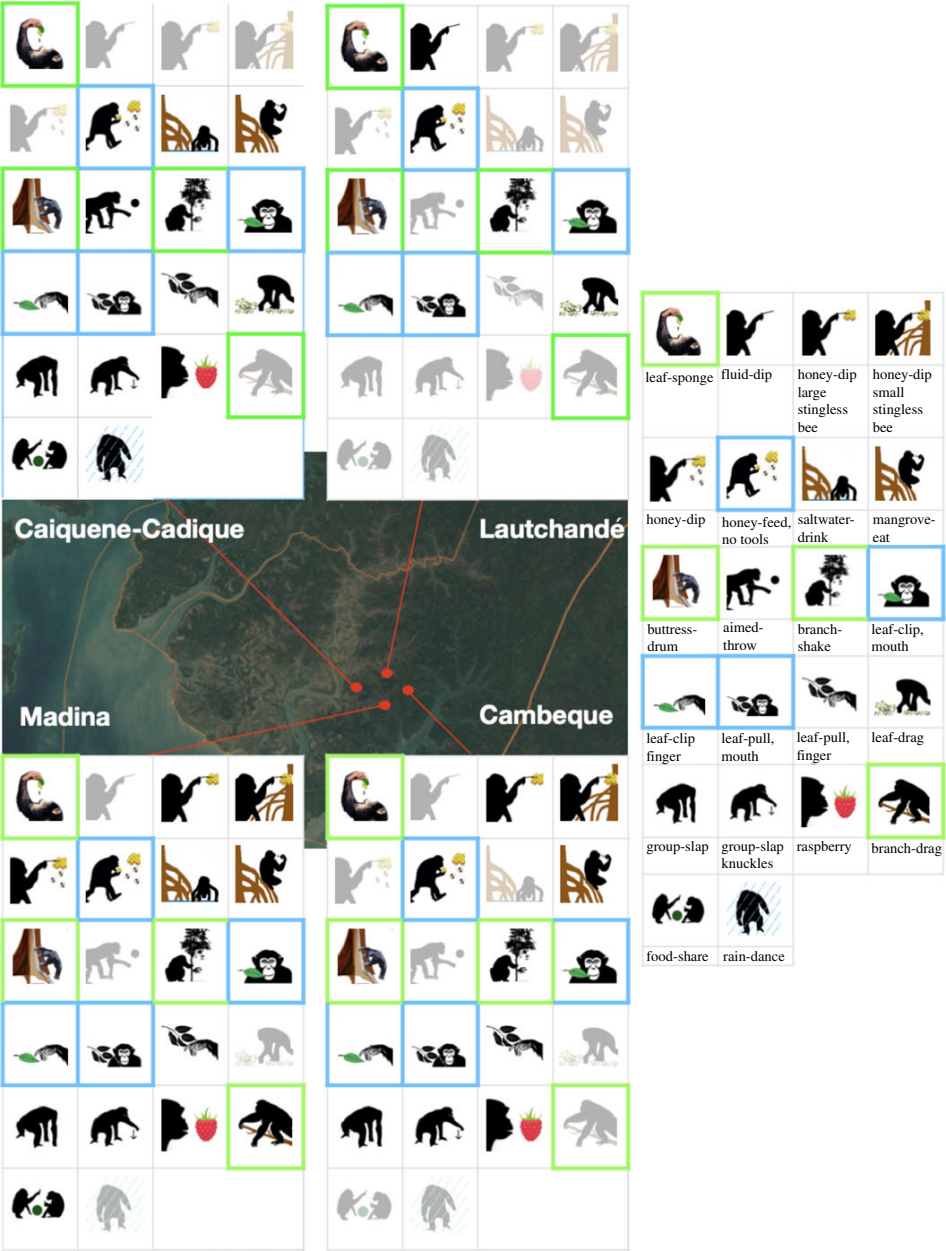
Distribution of behaviours across the four study sites (Caiquene-Cadique, Lautchandé, Madina and Cambeque). Coloured icons represent confirmed behaviours (C), and faded icons represent behaviours for which presence is still unconfirmed (U). See figure key for correspondence of icons to specific behaviours, described in detail in table 2. Green squares represent known species universals, and blue squares represent universals for the CNP study sites. The Sentinel-2 imagery was downloaded from the Sentinel Hub, Sinergise Ltd (https://www.sentinel-hub.com/). All maps were created using QGIS version 3.10.5 (https://www.qgis.org).

**Figure 3.**
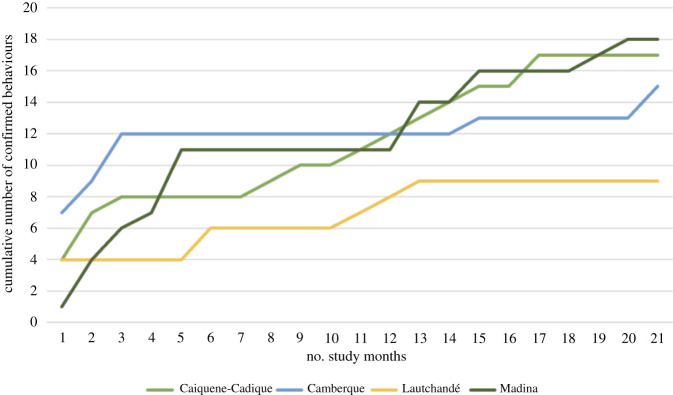
Cumulative number of confirmed behaviours found in each study community over the study period.

A total of 22 behaviours of interest were identified during the study period; figure 2 and table 1 show their distribution across the study communities, and figure 3 charts how their cumulative number varied with study effort (number of months of data collection). Four of these behaviours—Leaf-sponge, Buttress-drum, Branch-drag and Branch-shake—are universal for the species [25], while the remaining 18 behaviours are putatively cultural. Four of the putatively cultural behaviours (Leaf-clipping with mouth and with fingers, Leaf-pulling with fingers, and Honey consumption without the use of tools) appear to be universal for our four CNP study sites. Five other behaviours were only confirmed at one of the study sites: these included Rain-dance (Caiquene-Cadique), Aimed-throw (Caiquene-Cadique), Branch-drag (Madina), Honey-dip (Madina), Fluid-dip (Lautchandé). The site with the greatest number of behaviours confirmed was Madina (18), while Lautchandé had the fewest (9). The following sections describe variation in different behavioural domains across the four sites in more detail.

### 3.1. Extractive tool use

A total of 204 dipping tools and 16 leaf sponges were found across the CNP study sites. The pattern of tool recovery exhibited some marked variation among the four neighbouring communities. No evidence of extractive dipping tools to access fluid (including honey) was found in Caiquene-Cadique, while the extraction of different honey types with tools appeared to be frequent in Cambeque (100 tools) and Madina (103 tools) (table 1). Video evidence of extractive tool use was recorded in Madina and Cambeque.

### 3.2. Feeding and mangrove resource use

The use of mangroves and their resources (saltwater and salty leaves) was confirmed in three of the CNP communities. Chimpanzees at Caiquene-Cadique and Madina were seen Saltwater-drinking, and they, as well as Cambeque chimpanzees, were confirmed to chew on the salty leaves of black mangrove trees, *Avicennia germinans* (Mangrove-eat). No evidence of mangrove use was found at Lautchandé even though mangroves are present within the chimpanzees' known ranging area. Camera trap evidence of Mangrove-use was recorded for Madina and Caiquene-Cadique.

### 3.3. Communication and display

Leaf-clipping, a type of non-extractive tool use, was observed in all four CNP communities. All Leaf-clipping was confirmed through camera trapping and performed in association with Buttress-drumming. Leaf-pulling in association with drumming was also recorded in all CNP communities. Raspberry vocalization was captured on camera in three of the CNP communities, but not at Lautchandé. Additionally, in Caiquene-Cadique male chimpanzees were caught on camera throwing the giant fruit of *Treculia africana* during drumming displays.

### 3.4. Social behaviours

Fruit-sharing of large *T. africana* fruits was recorded on camera in Caiquene-Cadique and in Madina, between a mother and her dependent offspring as well as between adults. Rain-dance display was only confirmed at Caiquene-Cadique.

## 4. Discussion

An essential component of identifying cultural variation in non-human species is documenting behavioural variation across populations. However, studying behaviour and its variation in groups of unhabituated wild animals is a notoriously difficult and long process (e.g. [41]). Verifying the presence of specific behaviours and identifying inter-community variation are hampered by the patchy nature of available data, where the length of study and the methods employed have implications on the amount and nature of data that can be collected. Behaviours that leave behind material evidence, specific artefacts or modifications in the environment (e.g. feeding traces, tools, constructions) are easier to detect, while behaviours that leave no trace evidence (e.g. social or communicative behaviours) and/or are infrequent or rare in nature are impossible to document without direct *in situ* or remote observation. It is also evident that the longer a study runs the more researchers can learn about a population's behavioural repertoire, not simply because data accumulate over time but also because these studies may end up habituating or semi-habituating the individuals to researchers. Nonetheless, despite the constraints of working with unhabituated populations, the present study was able to compile, through a combination of direct and indirect methods, and remote monitoring, a list of behaviours and potential behavioural variations in four neighbouring chimpanzee communities in Guinea-Bissau. Furthermore, we were able to identify some noteworthy novel behaviours for these communities, as well as behaviours that are possibly new to the species as a whole, thus filling gaps in our knowledge of the chimpanzee behavioural repertoire at a continental scale.

We identified 22 distinct behaviours, some of which had previously been described as universal at a species level, such as Buttress-drumming and Leaf-sponging [17], and, therefore, will not be considered candidates for cultural behaviours. Even though we did not find all the universal behaviours in all the study communities we cautiously assume that this was an artefact of sampling time and methodology and that with increased study effort these will, in time, be identified for all communities. Several other behaviours appear universal at a local level in CNP such as Leaf-clipping or Honey-feeding (without tools), but when put into context at a subspecies or species level, are good candidates for cultural behaviour. This is either because the behaviour is known to be absent at least at some other study sites (e.g. Honey-feeding), the behaviour is exhibited in different contexts (e.g. Leaf-clipping), or there is as yet no evidence of the behaviour existing elsewhere (e.g. Mangrove-eat), suggesting variation across the species' range. Given our limitations (in terms of both data collection methods and study duration) it is not possible to conclusively confirm the *absence* of most of the studied behaviours, but considering the encounter rates of certain behaviours some putative variation seems likely. Perhaps the most striking case in point is Honey-dipping. While we confirmed the presence of at least one type of stingless bee (*Meliplebeia* sp.) in all four CNP communities’ ranges and the presence of honeybee (*Apis mellifera*) at all four study sites, evidence of dipping for honey was only found in two of the four CNP communities. Although we cannot conclude with certainty that dipping for honey is absent in Caiquene-Cadique and Lautchandé—and indeed the picture may change as research efforts continue—it is likely that it occurs with at least greater frequency in the communities of Cambeque and Madina (for further discussion see [40]). We chose to separate honey dipping into three types depending on the type of honey exploited (stinging bee, small stingless bee and large stingless bee honeys) since a previous study found significant differences in the number and types of tools used to extract these different types of honey [40]. We succeeded in confirming not only extractive tool use, but also non-extractive tool use. This type of behaviour does not produce recognizable artefacts and can only be documented through observation. Yet, despite working with unhabituated chimpanzees, we were able to remotely observe this behaviour through the use of camera traps. Leaf-clipping/pulling behaviours were confirmed in all CNP communities, and even though their function is as of yet unknown, they appear to play a role in Buttress-drumming displays. Hence, they could be considered examples of communicative or social tool use [51]. Additionally, there appears to be variation in Leaf-clipping and Leaf-pulling behaviours, and through further analyses we will be able to characterize these variations in more detail (manuscript in preparation). For example, in some cases a single leaf is carefully clipped, in others, several leaves are clipped simultaneously; on some occassions both varieties of leaf-clipping happen simultaneously (i.e. with mouth and hand) or leaf clipping and pulling are combined during the same event. Interestingly, such variation contrasts with what has been described at Tai Forest, the only other community known to Leaf-clip prior to drumming: there, according to Boesch [52], the behaviour is highly stereotyped among all the individuals of the group, unlike the within-community variation we observed. Also associated with buttress drumming we identified a Raspberry vocalization that is commonly heard in captivity and has been described as species atypical behaviour [53]. This behaviour has only been described in the wild once, in the Ngogo community (Uganda), but in the context of grooming [54].

One group of behaviours, which occurred exclusively in the mangrove habitat, is of particular interest. To our knowledge the use of mangroves has only been described in Loango (Gabon) [55], but see [56] for another potential example, although this study did not report whether chimpanzees used the mangroves within their habitat. Mangroves are an integral part of the Cantanhez landscape and evidence of chimpanzees utilizing resources within was confirmed for three of the four study sites. At the fourth, Lautchandé, we were not able to confirm any mangrove-related behaviours despite the presence of mangroves in the community's habitat. It may be the case that with the increase of study effort this will change; however, similarly to honey dipping, it is possible that different communities rely more on this habitat type than others. In the sense that mangrove-related behaviours are so clearly dependent on the environment, they can be compared to cave use by chimpanzees at other sites in West Africa (e.g. Fongoli in Senegal [31]). Ecology can easily explain the absence of the behaviour in many communities, yet it can still vary between communities where ecological characteristics alone cannot account for such variation, and, as such, cave use was considered a cultural behaviour by Kühl and colleagues [5]. This affirms the value of incorporating ecology and habitat in studies of behavioural variation, and illustrates why it is fruitful to explore new areas and habitat types when studying chimpanzee behaviour. In a similar vein, our findings regarding some less common social behaviours, such as Food-sharing and Rain-dance, detected in at least one of the studied communities, demonstrate the importance not only of study duration but also of diversification in the range of possible behaviours that researchers should look for in unhabituated communities.

It is worth noting that despite the study effort being similar across the four sites (table 1), the number of videos and indirect traces collected varied substantially between communities. This may in part have been due to the fact that at the beginning of our study we had a better baseline knowledge of the movements of the Caiquene-Cadique community than any of the other three communities, and hence were able to record a much larger number of chimpanzee videos at Caiquene-Cadique. However, this would not explain why in the first month of the study we in fact confirmed many more behaviours in Cambeque than in the other communities (figure 3). Additionally, the differences in ranging areas (with chimpanzees at Madina and Caiquene-Cadique estimated to range over larger areas than those at Cambeque and Lautchandé) could also help account for some of the disparity in number of indirect traces found: larger ranging areas may correspond to larger population sizes, which, in turn, may produce larger numbers of traces. It is likely that with an increase in study effort better parity in data volume across the four communities would be achieved, which may further clarify how much of the observed intercommunity variation found is due to behavioural differences.

Notwithstanding, in 2 years of research, we were able to compile an extensive list of wild chimpanzee behaviours never before described for CNP, for Guinea-Bissau, or (in the case of some behaviours) for the rest of Africa. We concede that this list is likely to be far from comprehensive since it is clear that longevity of study and close, direct observation of individuals are key factors that influence the size of the behavioural repertoire assembled for each chimpanzee community, as is evident through the increase in the number of confirmed behaviours over time (figure 3). The recent study by Kühl *et al.* [5] comparing the presence/absence of 31 behaviours among 144 chimpanzee communities (46 previously largely unstudied communities along with information from published literature on an additional 106 communities) concluded that these behaviours had on average 88% less chance of occurring in chimpanzee communities inhabiting highly anthropogenically impacted areas compared to low impacted areas [5]. This would thus seem to discourage the study of communities that, like ours, inhabit fragmented forest mosaics [57]. However, our results show that chimpanzee communities that inhabit human-impacted environments demonstrate rich behavioural repertoires. In CNP we were able to confirm 9 of the 65 behaviours described by Whiten *et al.* [25] and 7 of the 31 behaviours described by Kühl *et al.* [5]. These numbers might seem low, but given that the original lists only include, by definition, behaviours that show variation across communities, it is in fact expected that we will only encounter some of these behaviours in newly studied communities. Examining the data presented by Kühl *et al.* [5], even communities that are fully habituated and have been studied for over 40 years, only show a subset of the 31 behaviours analysed (e.g. 18 for Bossou, Guinea and 17 for Gombe, Tanzania).

Thus, our research shows that studying the behaviour of neighbouring primate communities inhabiting human-impacted areas can be a useful source of information in studies of animal culture, and re-affirms the value of using a combination of direct and indirect methodologies to document the behaviour of unhabituated communities. The fact that over the course of our relatively short study we identified behaviours which do not leave noticeable material evidence behind and that are rare, seasonal, or absent in other known chimpanzee communities, provides further justification for the validity of this approach. Additionally, and contrary to some previous studies, our approach was not constrained to a pre-selected list of behaviours. This allowed us to, for example, explore behaviours specific to unusual habitat types, such as mangroves, and identify previously unknown behaviours that we would have missed otherwise. At the same time, it is important to remember that behavioural repertoires are not static and should not be seen as such: behaviours can disappear [58], resurface after years of absence [59], change [32] or be, as far as we know, newly invented [23]. In fact, the ever-changing conditions that chimpanzees inhabiting disturbed habitats face might translate into a need to rapidly adapt through changes in established behaviours or through innovation [4]. This means that while some behavioural variants might disappear [5], given the flexible nature of chimpanzee behaviour others might arise anew and be passed down to the next generation via social learning [4]. Furthermore, human–chimpanzee dynamics are different in areas where local people have prolonged sympatry or exposure to wildlife and more gradually encroach into wildlife habitats, than those where human encroachment is more rapid and chimpanzees are killed for food or as ‘pests’ [4,60]. Thus, chimpanzees that for generations have been facing human disturbances and are tolerated by local people, such as our study communities, might display a rich behavioural repertoire that has allowed them not to become dependent on specific foods or habitat types (e.g. primary forest) to survive, and therefore might be better equipped for a continued existence in human-disturbed areas. In a related vein, it is increasingly evident that in addition to comprehensive and up-to-date information on the species' and subspecies’ status, distribution and population trends, genetic and cultural diversity are also important to guide effective conservation activities [4]. Our research helps build baselines for chimpanzee cultural diversity in CNP and for the species as a whole which has the potential—if done carefully [61]—to be integrated into existing, evidence-based conservation frameworks.

## Data accessibility

All camera trap data used for this research have been made accessible in the form of an Excel file uploaded as electronic supplementary information—electronic supplementary material, table S2.

The data are provided in electronic supplementary material [62].
